# A Review of Recent Advancements in Electrospun Anode Materials to Improve Rechargeable Lithium Battery Performance

**DOI:** 10.3390/polym12092035

**Published:** 2020-09-07

**Authors:** Byoung-Sun Lee

**Affiliations:** School of Polymer System/Department of Fiber Converged Material Engineering, College of Engineering, Dankook University, 152 Jukjeon-ro, Suji-gu, Yongin 16890, Korea; bslee2020@dankook.ac.kr; Tel.: +82-31-8005-3575

**Keywords:** electrospinning, intercalation anode, insertion anode, conversion anode, alloying anode, plating anode, lithium-ion battery, lithium metal battery

## Abstract

Although lithium-ion batteries have already had a considerable impact on making our lives smarter, healthier, and cleaner by powering smartphones, wearable devices, and electric vehicles, demands for significant improvement in battery performance have grown with the continuous development of electronic devices. Developing novel anode materials offers one of the most promising routes to meet these demands and to resolve issues present in existing graphite anodes, such as a low theoretical capacity and poor rate capabilities. Significant improvements over current commercial batteries have been identified using the electrospinning process, owing to a simple processing technique and a wide variety of electrospinnable materials. It is important to understand previous work on nanofiber anode materials to establish strategies that encourage the implementation of current technological developments into commercial lithium-ion battery production, and to advance the design of novel nanofiber anode materials that will be used in the next-generation of batteries. This review identifies previous research into electrospun nanofiber anode materials based on the type of electrochemical reactions present and provides insights that can be used to improve conventional lithium-ion battery performances and to pioneer novel manufacturing routes that can successfully produce the next generation of batteries.

## 1. Introduction

Lithium-ion batteries are a portable power source with a high energy density and stable electrochemistry that have changed our daily lives. Thanks to technological developments in areas such as smartphones and electric vehicles, there is an increased demand for high energy density and fast-charging lithium-ion batteries that can provide greater power capacity. Recent battery fires and explosions have also led to a desire to improve the safety of energy storage systems [1,2]. The design of a novel negative electrode material can address the energy density, safety, and rate performance issues of conventional graphite electrodes that cause unsatisfactory electrochemical performances such as low theoretical capacity (372 mAh/g) [3], irreversible electrolyte and lithium consumption based on solid electrolyte interphase (SEI) formation [4], slow lithium intercalation [5], and dendrite formation during fast charging [6]. Resolving these issues has been the focus of publications on novel lithium-ion battery anodes.

Despite positive results seen in the literature, conventional lithium-ion batteries still use graphite anodes. The two main factors preventing most novel technologies from being implemented in commercial lithium-ion batteries are (i) a need to reduce battery prices rapidly (targeting $125/kWh by 2022) [7] and (ii) a large number of specification requirements for commercial battery anode materials such as areal capacity (4 mAh/cm^2^) [8], electrode density (1.6 g/cm^3^) [9], electrode volume change (15%) [10], initial coulombic efficiency (90% to 95%) [11], and various target cycling and rate performance specifications dependent on purpose. Implementing major existing anode material research innovations into a commercial lithium-ion battery and designing novel anode materials and structures should be simultaneously pursued to meet the future demands for high energy density, high safety, and fast-charging lithium-ion batteries.

Electrospinning has been identified as the most promising route for designing novel anode materials and structures, owing to the simple process setup and wide variety of electrospinnable materials. The electrospinning process can encourage the implementation of existing anode material research based on the process being able to mass-produce anodes [12,13,14]. Although nanofiber anode material research has mainly focused on developing carbon-, silicon-, and tin-based materials to replace graphite anodes, there have been numerous publications on a wide variety of anode materials thanks to the merits of the electrospinning process. Here, examples to design the advanced anode materials based on the electrospun nanofibers are presented. Heteroatoms and pores were employed to increase the specific capacity of carbon anode [15,16]. Carbon composited and nanostructured metal and metal oxide anode materials were designed to improve cycling and rate performances [17,18]. Lithiophilic nanofiber was fabricated to enhance the reversibility of lithium plating and stripping [19]. It is necessary to review previous research into electrospun nanofiber-based anode materials to establish better strategies for implementing nanofiber anode materials in commercial lithium-ion batteries and designing novel nanofiber anode materials for next-generation batteries.

There have been two types of typical review articles on the electrospun nanofiber anode materials: (i) focusing on specific materials and structures (e.g., reviews of carbon, silicon, tin, and their composite nanofibers and nanostructures for lithium rechargeable batteries [20,21,22,23,24] and hollow, porous, and hierarchical structured nanofibers for energy applications [25,26,27]) and (ii) providing a broad overview of recent research and developments [28,29,30,31]. Regardless of the review types, the electrospun nanofiber anode materials in the previous review articles were mostly categorized into the material classes, for example, carbon, metals, and metal oxides, and so on [28,29]. The electrochemical behaviors of the anode materials are, however, not simply classified into the material classes as follows: TiO_2_ stores lithium by insertion, FeO_x_ is lithiated by conversion reaction, and SnO_x_ electrochemically mainly reacts with the lithium by alloying mechanism. In addition, some anode materials have multiple lithium storage mechanisms. As such, it is important to classify the anode materials into the categories of the main lithium storage principle for better understanding of the anode materials. This review focuses on the lithium storage principles and accordingly categorizes the electrospun nanofiber anode materials into the principles. This review aimed to provide inspiration to those working to improve conventional lithium-ion battery performances and pioneering novel routes to next-generation battery success.

## 2. Basic Lithium Storage Principles

**Figure 1:**
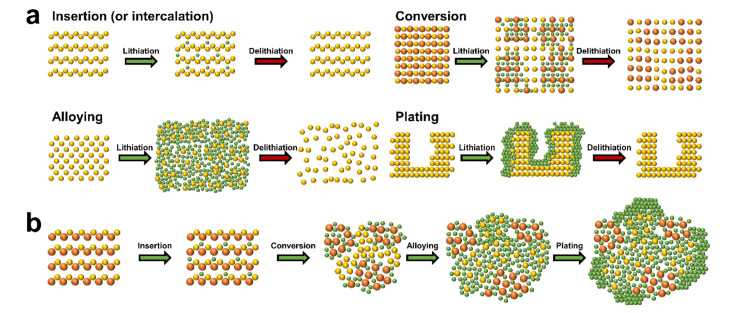
Schematic diagrams of (**a**) basic lithium storage principles of the anode materials and (**b**) microstructural change of ceramic materials during the stepwise lithiation processes.

It is crucial to understand the various mechanisms, advantages, and disadvantages of lithium storage in each anode material when designing high performance anodes. Figure 1a shows the anode materials of lithium rechargeable batteries categorized into four main groups based on electrochemical reactions. The first group is that of insertion (or intercalation) anode materials. Lithium-ions are reversibly inserted into and extracted from the periodic microstructure with little or no microstructural change resulting from the insertion/extraction (or intercalation/ deintercalation) mechanism. Representative insertion anode materials such as graphite and Li_4_Ti_5_O_12_ have been successfully commercialized based on their common strengths (e.g., small volume change during the electrochemical reaction and long lifespan based on the structural stability). However, low theoretical specific capacities (e.g., graphite (372 mAh/g) [32] and Li_4_Ti_5_O_12_ (175 mAh/g) [33]), which are the major drawback of insertion materials, have encouraged the development of alternative anode materials such as conversion and alloying anode materials. The second group is that of conversion reaction-based anode materials. These materials store lithium by altering the ionic bonding from a metallic cation and anion to a lithium-ion and anion in ionic compounds such as metal oxides, metal sulfides, and metal selenides. Microstructural changes that occur to the conversion anode materials during the electrochemical reactions separate the materials into small grains consisting of metal and lithium-anion complexes, which provide higher specific capacities in comparison with the insertion anode materials. However, poor kinetics, large volume changes, and the large redox potential hysteresis prevent commercialization of conversion anode materials. The third group is that of alloying anode materials that provide an increase in specific capacity. This is achieved when the lithium atoms form an alloy with the host metallic phase by breaking the inter-atomic bonds of the host material. However, the large volume changes driven by the insertion and extraction of the large amount of lithium cause pulverization, solid electrolyte interphase (SEI) growth, and electrical contact loss. The fourth group is that of plating anode materials. A vast amount of lithium can be stored by the plating mechanism as the lithium is stored in a free volume. The lithium-ions are deposited on the inactive lithium surface by electrochemical reduction and the lithium atoms are stripped from the surface by electrochemical oxidation. However, critical issues such as dendrite growth, severe SEI growth, and dead lithium formation need to be addressed before plating anode materials can be incorporated into commercial lithium rechargeable batteries.

The electrochemical behaviors of single element anode materials, such as graphite and crystalline silicon, can be mostly understood from the lithiation mechanisms. For example, lithium storage in artificial graphite is achieved using insertion and plating mechanisms [34]. However, the lithiation behaviors of ceramic anode materials consisting of multiple elements are very complex because of the multiple electrochemical reactions. For instance, tin oxide (SnO_2_) is lithiated via insertion (SnO_2_ + xLi^+^ + xe^−^ ↔ Li_x_SnO_2_), irreversible conversion (SnO_2_ + 4Li^+^ + 4e^−^ → Sn + 2Li_2_O), and alloying (Sn + xLi^+^ + xe^−^ ↔ Li_x_Sn (0 ≤ x ≤ 4.4)) [35]. When focusing on the microstructural change of specific regions during lithiation, the lithiation mechanism follows a sequential order. The schematic showing the microstructural changes of a virtual ceramic material is shown in Figure 1b to show the sequential order of lithiation: (i) a small amount of lithium atoms stored in the lattice from insertion (or intercalation), (ii) the lithium-ions are bonded to the anions by altering the reduced metallic cations through conversion, (iii) the lithium atoms form an alloy with the fully reduced metallic atoms via alloying, and (iv) the lithium-ions are reduced and deposited on the electrically conductive surface of the ceramic material owing to the fully lithiated microstructure being inactive.

## 3. Insertion/Extraction (or Intercalation/Deintercalation)-Based Storage Materials

Graphite and Li_4_Ti_5_O_12_ are very well-known intercalation/deintercalation-based storage materials that are used as anode materials in commercial lithium rechargeable batteries. Graphite has been widely used in various applications thanks to its theoretical capacity of 372 mAh/g and low average working potential of ca. 0.2 V [36]. The kinetics during the electrochemical reaction are limited because lithium-ions can only be intercalated and deintercalated through the inter-basal planes in the anisotropic graphite microstructure. Lithium dendrites are prone to formation on the graphite surface as a result of the low working potential [37]. Moreover, the low theoretical capacity in comparison with silicon-based materials and lithium metal anodes has also been recognized as a drawback of graphite anodes [38].

**Figure 2:**
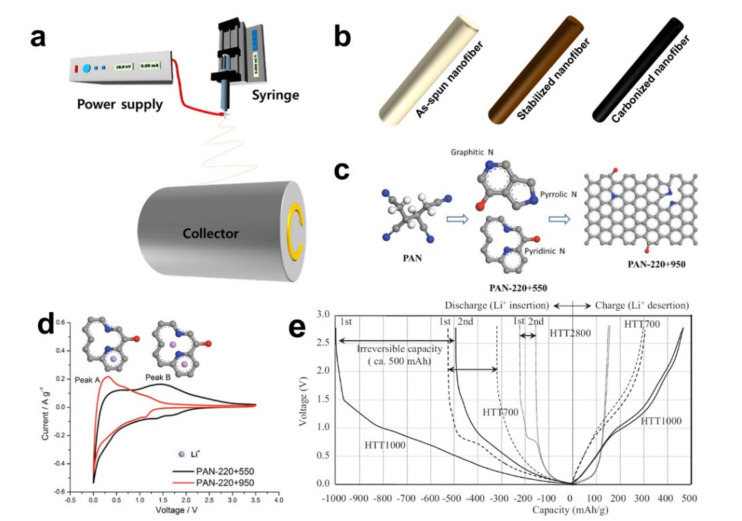
Schematic diagrams of (**a**) electrospinning process, (**b**) morphological changes of nanofibers during thermal treatment, and (**c**) proposed atomic arrangements. Reprinted with permission from [41]. Copyright 2013 John Wiley and Sons. Electrochemical characterization results of the carbon nanofibers: (**d**) cyclic voltammograms (CVs) of the carbon nanofibers carbonized ad 550 and 950 °C. Reprinted with permission from [41]. Copyright 2013 John Wiley and Sons. (**e**) Voltage profiles of carbon nanofibers carbonized at 700, 1000, and 2800 °C. Reprinted with permission from [39]. Copyright 2006 John Wiley and Sons. PAN, polyacrylonitrile.

Electrospinning and subsequent heat treatment of carbon-based nanofibers (CNFs), shown in Figure 2a,b, have been proposed as a novel anode material owing to the nanosize effect [39], the tunable microstructure [40], and the inherent presence of heteroatoms [41]. Polyacrylonitrile (PAN) is the most extensively used precursor because of its well-established fabrication process. Figure 2c shows a representation of the atomic arrangement of the PAN precursor and the changes during the thermal treatment. The PAN molecules form a basal plane with a reduced number of heteroatoms (e.g., inherently existing nitrogen and newly introduced oxygen during oxidative stabilization) as the heat treatment temperature increases. Cyclic voltammograms (CVs) of the CNFs carbonized at 550 and 950 °C are shown in Figure 2d and the graph shows two oxidation peaks at 0.3 (peak A of the CNF carbonized at 950 °C) and 1.5 V (peak B of the CNF carbonized at 550 °C) [41]. Peak A was attributed to the delithiation of the lithium-ions between the graphene layer, while peak B resulted from the extraction of the lithium-ions from the defects created by the nitrogen atom in the basal plane. The presence of a heteroatom not only contributes to the change of the working potential, but also increases the specific capacity by providing more lithium storage sites. Figure 2e compares voltage profiles of the initial cycles of CNFs carbonized at 700, 1000, and 2800 °C [39]. Both the CNFs carbonized at 700 and 1000 °C show the sloppy voltage profiles with subtle plateaus and higher specific capacities of 510 and 1000 mAh/g than that of the theoretical value, but their initial coulombic efficiencies were rather low owing to the SEI formation at around 0.8 V. The graphitized CNF that was carbonized at 2800 °C showed a typical graphitic intercalation voltage profile with a low specific capacity of 130 mAh/g. In brief, the CNFs have shown highly tunable electrochemical performances in accordance with their fabrication process.

**Figure 3:**
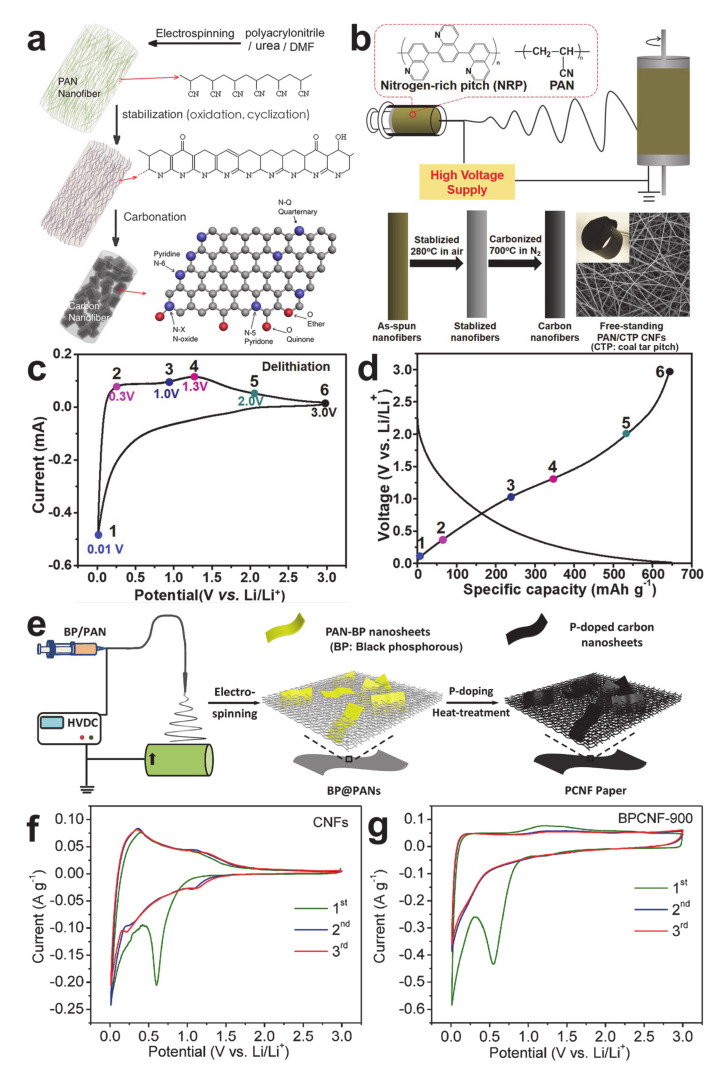
Heteroatom-doped carbon nanofiber anode materials. Schematic diagrams of nitrogen-doped carbon nanofibers fabrication processes based on (**a**) urea (reprinted with permission from [42]; copyright 2016 American Chemical Society), (**b**) nitrogen-rich pitch (reprinted with permission from [43]; copyright 2018 Elsevier), and the electrochemical performances of the nitrogen-doped carbon nanofibers: (**c**) cyclic voltammogram and (**d**) voltage profile (reprinted with permission from [43]; copyright 2018 Elsevier). (**e**) Schematic diagram of the phosphorous-doped carbon nanofiber fabrication process, and cyclic voltammograms of (**f**) pristine and (**g**) phosphorous-doped carbon nanofibers. Reprinted with permission from [44]. Copyright 2018 Elsevier. CNF, carbon-based nanofiber.

The effect of a heteroatom has been further pursued by introducing sources of heteroatoms. Urea and nitrogen-rich pitch (NRP) were employed as nitrogen sources to design nitrogen-doped CNFs with high specific capacities, as shown in Figure 3a,b [42,43]. The CV and voltage profile of the nitrogen-doped CNFs in Figure 3c and d reveal both the intercalation mode (below 1 V, corresponding to peak A in Figure 2d) and adsorption mode driven by the nitrogen atoms (above 1 V, corresponding to peak B in Figure 2d). Note that the reversible capacity of the nitrogen-doped CNFs was mainly attributed to the adsorption mode. Phosphorous-doped CNFs were prepared by adding black phosphorus to the electrospinning dope, as shown in Figure 3e [44]. The CV of the pristine CNFs shown as a control group in Figure 3f reveals the intercalation-based lithium storage. The electrochemical behavior of the phosphorous-doped CNFs in Figure 3g is analogous to that of the nitrogen-doped CNFs. It is clear that the presence of heteroatoms contributes to the increased specific capacity of the CNFs owing to the adsorption mechanism at high potentials (above 1 V), but the capacity improvements from >1.5 V may not be useful in practical applications, as full cells are usually cycled in narrower potential windows (≤1.5 V) (e.g., between 2.75 and 4.2 V [45] and between 3.0 and 4.4 V [46]).

**Figure 4:**
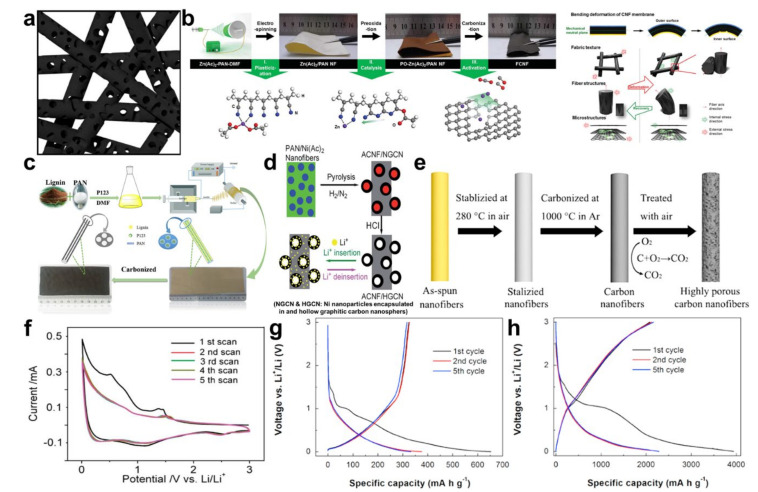
Porous carbon nanofiber anode materials. (**a**) Schematic illustration of porous carbon nanofibers (reprinted with permission from [48]; copyright 2009 Elsevier) and various porous carbon nanofibers based on (**b**) zinc precursor (reprinted with permission from [50]; copyright 2017 Royal Society of Chemistry), (**c**) triblock copolymer Pluronic P 123 (reprinted with permission from [51]; copyright 2017 Elsevier), (**d**) Ni nanoparticles (reprinted with permission from [37]; copyright 2012 Royal Society of Chemistry), and (**e**) partial oxidation at high temperature (reprinted with permission from [53]; copyright 2015 Elsevier). Electrochemical characterization results of the porous carbon nanofibers: (**f**) cyclic voltammograms of Ni nanoparticle-based porous carbon nanofibers (reprinted with permission from [37]; copyright 2012 Royal Society of Chemistry) and voltage profiles of (**g**) carbon nanofibers and (**h**) porous carbon nanofibers (reprinted with permission from [53]; copyright 2015 Elsevier).

Pores in carbon microstructures have also been considered as a mechanism for increasing the storage capacity of lithium-ions [47]. As such, various types of porous carbon nanofibers (Figure 4a) have been proposed using various pore generating materials. SiO_2_ nanoparticles and precursors were employed as a mesopore templating material [48,49], while a zinc precursor (Zn(Ac)_2_) was used for generating micropores via an oxidizing chemical reaction (ZnO + C → Zn↑ + CO_x_↑) in the CNFs (see Figure 4b) [50]. Thermally decomposable polymers such as triblock copolymer Pluronic P 123, shown in Figure 4c, have been actively introduced as a mesopore generating template [51]. As shown in Figure 4d, transition metallic precursors (e.g., Ni(Ac)_2_) were added in the spinning dope to form metallic nanoparticles in the heat treatment phase, and the nanoparticles were leached out using acid to leave the mesopores [37]. The activation of CNFs can produce intensive micropores in the carbonaceous microstructure [52]. Likewise, thermal oxidation during the carbonization process in Figure 4e is one of the most efficient means to develop micropores [53]. Figure 4f shows the typical CVs of the porous CNFs. The irreversibly developed peaks in the first reduction process are related to the SEI formation and irreversible intercalation to the mesopores and defects [54,55]. The redox peaks developed in the high voltage region (above 1 V) are still observed in subsequent cycles. The charging behaviors of the pristine CNF and the porous CNF electrodes in the voltage profiles (Figure 4g,h) have clear differences: the reversible capacity contribution from the CNF can mostly be attributed to the carbonaceous microstructure at a low potential range (≤1 V), while it is mainly a result of faradaic capacitance on the surface and large pore volumes at a high potential range (>1 V) [53]. The electrochemical behaviors of the porous CNFs are highly similar to those of the heteroatom-doped CNFs and the strengths and weaknesses of the porous CNFs are close to those of the heteroatom-doped CNFs.

**Figure 5:**
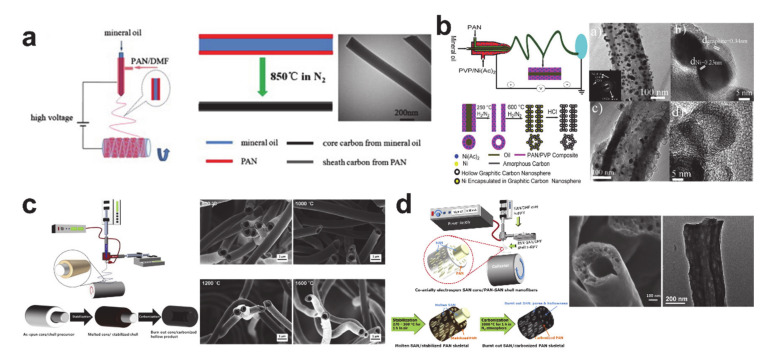
Coaxially electrospun carbon nanofiber anode materials. Schematic processing diagrams and resulting anode materials: (**a**) carbon/carbon core–shell nanofibers (reprinted with permission from [59]; copyright 2011 Elsevier), (**b**) hollow carbon nanosphere decorated hollow carbon nanofibers (reprinted with permission from [38]; copyright 2012 Royal Society of Chemistry), (**c**) hollow carbon nanofibers carbonized at various carbonization temperatures (reprinted with permission from [60] and [40]; copyright 2015 and 2012 Elsevier), and (**d**) mesoporous hollow carbon nanofibers (eprinted with permission from [54]; copyright 2012 American Chemical Society).

Coaxial electrospinning processes enable complicated designs of the CNFs from tubular or core–shell structures to the wire-in-tube and tube-in-tube structures via fabricating bi-, tri-, and tetra-layered precursor nanofibers [56,57,58]. As such, there is scope to improve the electrochemical performance of the CNFs. Selectively placing different quality carbon in the core and on the shell in Figure 5a was achieved using the coaxial electrospinning process with a mineral oil core as a soft carbon source and the PAN shell as a hard carbon source [59]. Interestingly, the mineral oil was also used as pore generating material to form hollow CNFs, as shown in Figure 5b [38]. The porous structure of the hollow CNFs was attributed to the thermally decomposed polyvinyl pyrrolidone (PVP) and leached out Ni nanoparticles. The porous hollow CNFs in Figure 5b demonstrated excellent electrochemical performances that can be attributed to their porous microstructure.

The hollow CNFs are a very useful platform material for designing advanced composite anode materials with conversion or alloying anode materials. However, the use of mineral oil or an improper core material can cause instabilities in the electrospinning process. It is thus very important to use the appropriate core material. The use of an acrylonitrile copolymer (e.g., styrene-*co*-acrylonitrile (SAN)) is beneficial when forming a stable hollow structure, owing to its compatibility with PAN precursor and thermal degradability. Well-defined hollow CNFs and porous hollow CNFs were fabricated using the SAN core–PAN shell solution combination (see Figure 5c,d) [40,54,60]. The microstructure and electrochemical performances of the hollow CNFs were similar to those of the solid CNFs, while those of the porous hollow structures were analogous to those of the porous CNFs. Thus, the main contribution to the improved capacity of the CNFs ensues from the heteroatom and porous structures.

Though novel insertion (or intercalation) anode materials such as TiNb_2_O_7_ [61,62], N_2_O_5_ [63], Li_2_ZnTi_3_O_8_ [64], and BaLi_2_Ti_6_O_14_ [65] have been actively explored, it is mostly traditional titanium-based oxide materials (TiO_2_ and Li_4_Ti_5_O_12_) that have been highlighted as promising insertion anode materials. TiO_2_ is an established anode material that has a relatively high working potential of ca. 1.7 V and theoretical capacity of 335 mAh/g (TiO_2_ + Li^+^ + e^−^ ↔ LiTiO_2_) [66,67,68], while Li_4_Ti_5_O_12_ has a working potential of 1.55 V and theoretical capacity of 175 mAh/g (Li_4_Ti_5_O_12_ + 3Li^+^ + 3e^−^ ↔ Li_7_Ti_5_O_12_) [69,70,71,72]. Titanium-based anode materials are attractive because they suppress dendritic lithium growth and SEI formation, a result of the high working potentials of TiO_2_ and Li_4_Ti_5_O_12_ [73,74,75]. As such, excellent cycling stability and high rate capability have been achieved with the design of nanofiber electrodes, and full cells based on the nanofiber electrodes exhibited excellent electrochemical performance [61,68,75,76].

**Figure 6:**
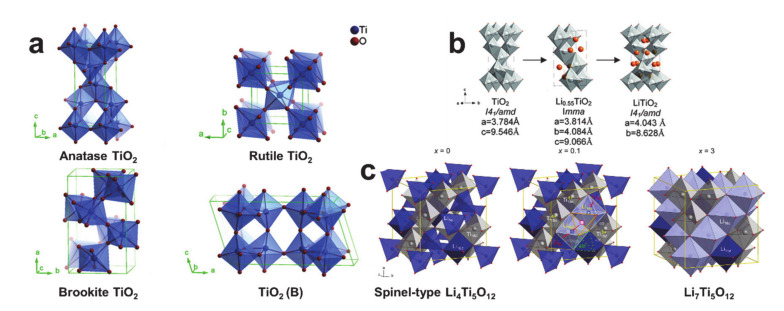
Crystalline structures and phase transition by lithiation of TiO_2_ and Li_4_Ti_5_O_12_. (**a**) Crystalline structures and TiO_2_ in different phases: anatase, rutile, brookite, and TiO_2_(B) (reprinted with permission from [77]; copyright 2014 American Chemical Society), (**b**) phase transition upon lithium insertion in anatase TiO_2_ (reprinted with permission from [78]; copyright 2010 American Chemical Society), and (**c**) crystalline structural changes of spinel-type Li_4_Ti_5_O_12_ as lithium occupation increases (reprinted with permission from [79]; copyright 2015 American Chemical Society).

It is necessary to understand the crystalline structures of the titanium-based anode materials before studying the titanium-based nanofiber anode materials. Figure 6 shows the crystalline structures and the phase transition of TiO_2_ and Li_4_Ti_5_O_12_ [77,78,79]. TiO_2_ has eleven polymorphic phases including anatase (tetragonal, *I*4_1_/amd), rutile (tetragonal, *P*4_2_/mnm), brookite (orthorombic, *P*bca), TiO_2_(B)-bronze (monoclinic, *C*2/m), TiO_2_(H)-hollandite, (tetragonal, *I*4/m), and TiO_2_(R)-ramsdellite (orthorombic, *P*bnm) [80]. The anatase, rutile, brookite, and TiO_2_ (B) phases shown in Figure 6a are known to be electrochemically active with lithium [74]. The rutile phase is thermodynamically stable, while the anatase and brookite phases are metastable. Despite it being metastable, the anatase is the most effective crystalline phase to store lithium, owing to its open crystal structure, a fact that has also been proven using electrospun TiO_2_ nanofibers [81]. As shown in Figure 6b, the anatase phase experiences two phase transitions: TiO_2_ (*I*4_1_/amd) changing into Li_0.55_TiO_2_ (*I*mma) and subsequently transforming into Li_1_TiO_2_ (*I*4_1_/amd) [78]. Though the pristine and fully-lithiated crystalline structures of the anatase TiO_2_ are the same, the electrochemical reaction causes a volume change of 3% [82]. The spinel-type Li_4_Ti_5_O_12_ is transformed to rock-salt type Li_7_Ti_5_O_12_, as shown in Figure 6c [79]. During lithiation, the original lithiums at the 16d and 8a positions of the spinel-type Li_4_Ti_5_O_12_ are redistributed when insertion of the lithium ions occurs and the Ti^4+^ ions are partially reduced to Ti^3+^. Despite the significant crystallographic change during the electrochemical reactions, the spinel-type Li_4_Ti_5_O_12_ is known as a “zero-strain” material [73]. Thus, choosing between the titanium-based anode materials of TiO_2_ and Li_4_Ti_5_O_12_ is a matter of selecting the desired beneficial property (i.e., the high specific capacity of TiO_2_ or the zero-strain of Li_4_Ti_5_O_12_).

**Figure 7:**
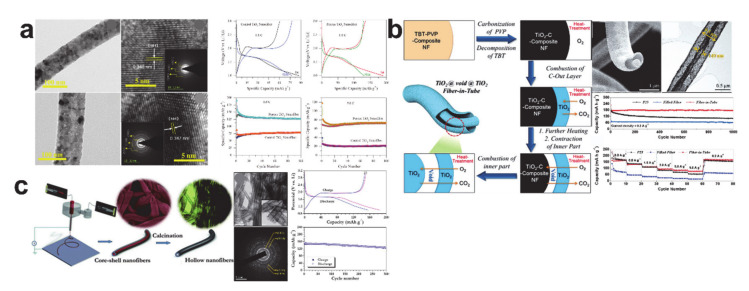
Nanostructured TiO_2_ anode materials and electrochemical performances. (**a**) Control and porous TiO_2_ nanofiber (reprinted with permission from [87]; copyright 2017 Royal Society of Chemistry), (**b**) fiber-in-tube structured TiO_2_ nanofiber (reprinted with permission from [88]; copyright 2015 John Wiley and Sons), and (**c**) hollow TiO_2_ nanofiber (reprinted with permission from [89]; copyright 2013 Royal Society of Chemistry). TBT, tetra-*n*-butyl titanate; PVP, polyvinyl pyrrolidone.

The critical challenges associated with TiO_2_ anode materials are mainly related to the kinetic nature (i.e., low lithium-ion diffusion rate (*ca.* 10^−15^ − 10^−9^ cm^2^/s) and inferior electrical conductivity (*ca.* 10^−12^ − 10^−7^ S/cm)) [66,83,84,85,86]. Designing one-dimensional nanostructures through the electrospinning process is considered an efficient route to overcome the diffusion issue related to the reduced lithium-ion diffusion path and a high surface-to-volume ratio [84]. Figure 7 shows the representative TiO_2_ one-dimensional nanostructures. Porous TiO_2_ nanofibers, which were prepared by adding a surfactant (cetyltrimethylammonium bromide (CTAB)) to the electrospinning dope (Figure 7a), exhibited a higher specific capacity and better cycling performances when compared with the control TiO_2_ nanofiber, owing to a shorter lithium-ion diffusion length and abundant channels for lithium ion transport [87]. A fiber-in-tube structured TiO_2_ nanofiber was prepared by a single-pot heat treatment process comprising of tetra-*n*-butyl titanate (TBT)–PVP composite nanofiber heated to 450 °C in a vessel containing pure oxygen through the illustrated mechanism in Figure 7b [88]. The key requirement in preparing the fiber-in-tube structured TiO_2_ nanofiber was burning the TiO_2_-C nanofiber for a short period under oxygen. The cycling performance of the fiber-in-tube structured TiO_2_ nanofiber was much better than the filled (controlled) TiO_2_ nanofiber, but the improved performance was assumed to be down to the differing crystalline structures (i.e., anatase phase of the fiber-in-tube structure and rutile phase of the filled structure). Hollow TiO_2_ nanofiber was synthesized using coaxial electrospinning of a polyethylene oxide (PEO) core/titanium tetraisopropoxide (TIP)–PVP shell solution combination following calcination [89]. The hollow TiO_2_ nanofiber with anatase phase exhibited a fair reversible capacity of ca. 150 mAh/g (equivalent to 0.45 moles lithium in Li_x_TiO_2_) and an excellent cycling retention of 84% after 300 cycles.

There have been efforts to improve the electrical conductivity of the TiO_2_ nanofibers. It is simple and intuitive to composite the TiO_2_ nanofibers with conducting materials such as gold or silver nanoparticles [83,90], reduced graphene oxide (rGO) [66,84], and hard carbon [85]. By extension, silver paste has been used to adhere the TiO_2_ active materials and current collector and to provide the electrical conducting pathway to the active materials [91], and the surfaces of the hollow TiO_2_ nanofibers were nitridated to increase electrical conductivity [92]. Niobium was doped into the TiO_2_ nanofibers to improve the electrical conductivity of the TiO_2_ by modifying the electronic properties to exhibit a metallic-type conduction behavior [86].

**Figure 8:**
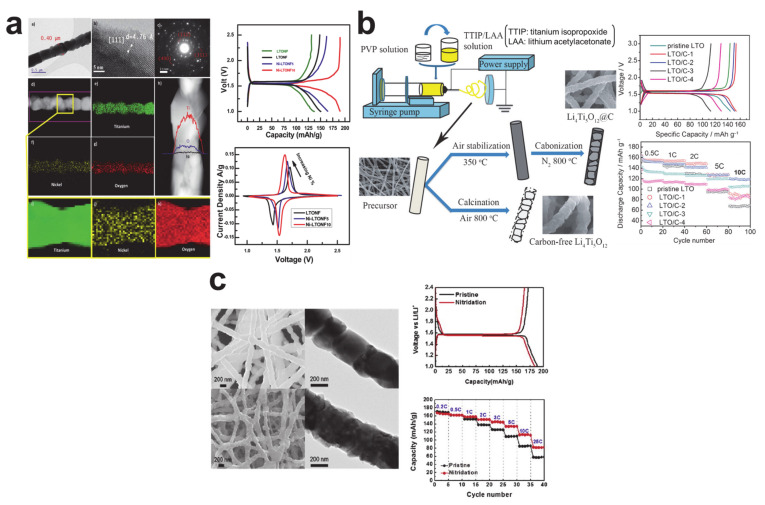
Li_4_Ti_5_O_12_ nanofiber anode materials and their electrochemical performances. (**a**) Nickel-doped Li_4_Ti_5_O_12_ nanofiber (reprinted with permission from [71]; copyright 2016 Royal Society of Chemistry), (**b**) Li_4_Ti_5_O_12_–carbon composite nanofiber (reprinted with permission from [95]; copyright 2013 John Wiley and Sons), and (**c**) nitridated Li_4_Ti_5_O_12_ nanofiber (reprinted with permission from [94]; copyright 2013 Elsevier).

Unlike the TiO_2_ anode material, issue of the Li_4_Ti_5_O_12_ anode materials is limited by poor electrical conductivity (<10^−13^ S cm^−1^) because of its excellent lithium-ion diffusion rate (2 × 10^−8^ cm^2^/s) [93,94]. As such, the efforts to improve the electrochemical performances of the Li_4_Ti_5_O_12_ nanofiber anode materials have been mainly focused on improving the electrical conductivity. As shown in Figure 8, the electrical conductivities of the Li_4_Ti_5_O_12_ were improved by doping nickel [71], compositing with a carbon matrix [95], and forming a nitridated layer [94]. Every single approach exhibited improved electrochemical performances by increasing the electrical conductivity. Likewise, doping of metallic ions (e.g., silver [96], niobium [97], zirconium [98], and chromium ions [99]) provided an electrochemical performance improvement in the Li_4_Ti_5_O_12_ nanofibers.

## 4. Conversion Reaction-Based Storage Materials

Environmentally friendly, nontoxic, abundant, and cost-effective transition metal oxides such as Fe_2_O_3_, Fe_3_O_4_, Co_3_O_4_, NiO, and MnO have attracted the interest of battery researchers for their high specific capacities in the conversion reaction. Electrochemical reactions of MO_x_ (where M is Mn, Fe, Co, and Ni) with lithium-ions can be generalized with respect to the stoichiometry factor x as follows: (i) MO: one mole of MO reacts to two moles of lithium-ions and produces one mole of M and one mole of Li_2_O (MO + 2Li^+^ + 2e^–^ ↔ M + Li_2_O); (ii) M_3_O_4_: one mole of M_3_O_4_ and eight moles of lithium-ions react to form three moles of M and four moles of Li_2_O (M_3_O_4_ + 8Li^+^ + 8e^–^ ↔ 3M + 4Li_2_O); (iii) M_2_O_3_: one mole of M_2_O_3_ reacts with six moles of lithium-ions to form two moles of M and three moles of Li_2_O (M_2_O_3_ + 6Li^+^ + 6e^–^ ↔ 2M + 3Li_2_O); and (iv) MO_2_: one mole of M and two moles of Li_2_O are formed by the reaction of one mole of MO_2_ and four molar lithium-ions (MO_2_ + 4Li^+^ + 4e^–^ ↔ M + 2Li_2_O). As such, specific capacities of the MO_x_ are increased as the stoichiometric factor x increases and the atomic number of M decreases as follows: MnO (755 mAh/g) [100,101], FeO (746 mAh/g) [102], NiO (718 mAh/g) [103,104], Mn_3_O_4_ (936 mAh/g) [105,106], Fe_3_O_4_ (924 mAh/g) [107,108], Co_3_O_4_ (892 mAh/g) [109,110], Fe_2_O_3_ (1007 mAh/g) [111,112,113], and MnO_2_ (1230 mAh/g) [114,115].

Despite the high specific capacities, commercialization of the transition metal oxide anode materials has so far proven difficult owing to the pulverization and electrical contact loss caused by the considerable microstructural rearrangement and large volume change during the electrochemical reaction. The design of nanostructured transition metal oxides has been widely employed to both accommodate the volume changes and reduce the lithium-ion diffusion pathways. One-dimensional nanostructured transition metal oxides and their derivatives created by the electrospinning process have been intensively researched for their morphological benefits (e.g., a high surface-to-volume ratio, short diffusion length, and axially connected electronic pathways [116,117,118]). The transition oxide nanofibers are generally synthesized by electrospinning a precursor solution followed by a calcination process performed under air. Though the one-dimensional nanostructure itself can withstand the electrochemical reaction driven mechanical stress [119], hollow structured transition metal oxides are more effective conversion anode materials over the solid (or filled) transition metal oxide nanostructures as the voids present are more able to accommodate the mechanical stress resulting from the volume change [120,121]. Template-free hollow nanostructured transition metal oxides have been mostly fabricated via Kirkendall diffusion (i.e., nonequilibrium diffusion) and inside-out Ostwald ripening (i.e., inhomogeneous particle growth by merging small particles) during thermal oxidation [122,123,124].

**Figure 9:**
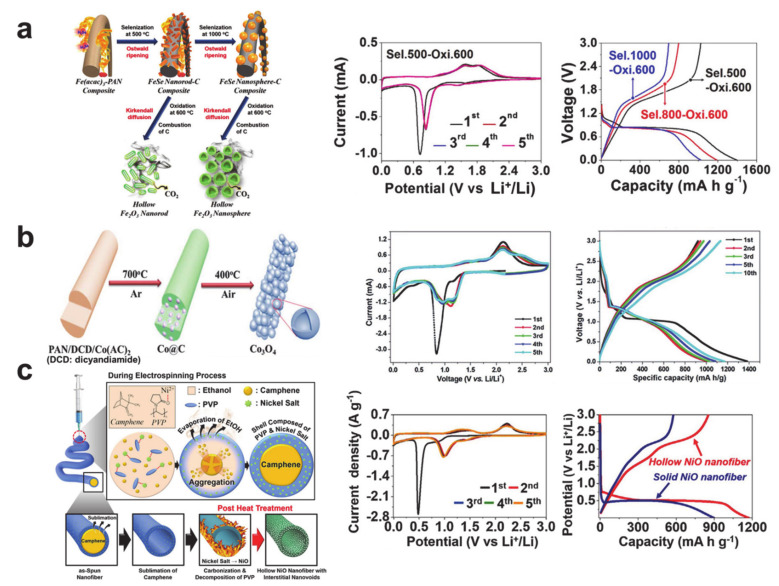
Schematic illustrations of hollow nanostructured transition metal oxide anode materials and corresponding electrochemical performances. (**a**) Fe_2_O_3_ nanostructures (reprinted with permission from [125]; copyright 2016 Springer Nature), (**b**) hollow Co_3_O_4_ nanostructure (reprinted with permission from [127]; copyright 2019 Royal Society of Chemistry), and (**c**) hollow NiO nanofiber (reprinted with permission from [130]; copyright 2019 Elsevier).

Hollow nanostructured transition metal oxide anode materials and their corresponding electrochemical performances are presented in Figure 9. Hollow Fe_2_O_3_ nanorods and nanospheres in Figure 9a were synthesized via selenization of a precursor nanofiber and a subsequent oxidation reaction [125]. The hollow nanorod exhibited typical CVs of Fe_2_O_3_ and the following electrochemical reactions: (i) lithium insertion at around 1.6 and 1V to form Li_2_Fe_2_O_3_ (cubic), the complete reduction of iron to Fe^0^, and Li_2_O formation/SEI formation at around 0.7 V during the first cathodic scan; (ii) the oxidation of iron from Fe^0^ to Fe^2+^ at 1.7 V and Fe^3+^ at 1.85 V during anodic scans; and (iii) the reduction of iron from Fe^3+^ to Fe^0^ at around 0.8 V during the following cathodic scans [120,126]. The relatively excellent performance of the one-dimensional structure was proven in the charge and discharge capacity differences seen in the hollow nanorod and nanosphere, which was determined by the selenization temperature. Figure 9b represents the interconnected hollow Co_3_O_4_ nanofiber prepared via electrospinning and subsequent annealing [127]. The CV curves exhibited the electrochemical reactions from Co_3_O_4_ to the lithium-ions as follows: (i) lithium insertion at around 1.1 V to form Li_x_Co_3_O_4_, complete reduction of cobalt to Co^0^, and Li_2_O formation/SEI formation at around 0.8 V during the first cathodic scan; (ii) the oxidation of cobalt from Co^0^ to Co^2+^/Co^3+^ to form Co_3_O_4_ at around 2.1 V during anodic scans; and (iii) the reduction of cobalt from Co^3+^/Co^2+^ to Co^0^ at around 1.15 and 0.95 V during the following cathodic scans [127,128,129]. Interestingly, the reversible capacity of the interconnected hollow Co_3_O_4_ nanofiber increased as the cycle number increased. The hollow NiO nanofiber in Figure 9c was also prepared via single nozzle electrospinning and a subsequent calcination process, but the Kirkendall diffusion behavior was designed with a vapor pressure difference between the organic molecules (ethanol and camphene) [130]. The CV curves revealed the cathodic and anodic reactions of NiO with the lithium-ions: (i) lithium insertion at around 0.63 V (shoulder peak) to form Li_0.5_NiO, the complete reduction of nickel to Ni^0^, and Li_2_O formation/SEI formation at around 0.5 V during the first cathodic scan; (ii) the oxidation of nickel from Ni^0^ to Ni^2+^ to form NiO at around 2.2 V during anodic scans; and (iii) the reduction of nickel from Ni^2+^ to Ni^0^ at around 1.2 V during the following cathodic scans [103,131,132]. It should be noted that there is a common irreversible insertion prior to the reversible conversion reactions of the transition metal oxides.

**Figure 10:**
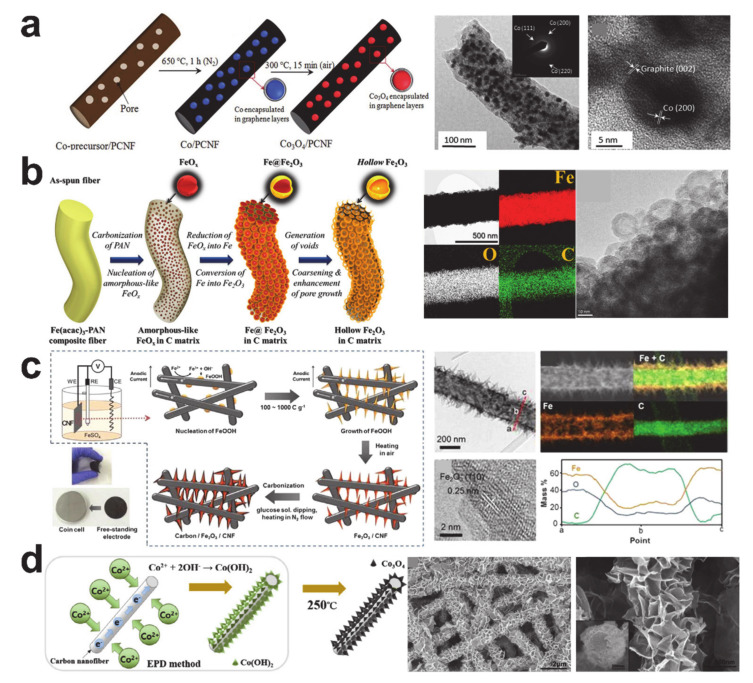
Transition metal oxide/carbon composite nanofibers. (**a**) Co_3_O_4_/porous carbon nanofiber (reprinted with permission from [109]; copyright 2014 Royal Society of Chemistry), (**b**) hollow Fe_2_O_3_/carbon composite nanofiber (reprinted with permission from [116]; copyright 2015 American Chemical Society), (**c**) Fe_2_O_3_ nanorods on carbon nanofiber (reprinted with permission from [112]; copyright 2015 Elsevier), and (**d**) flower-like Co_3_O_4_ nanosheets on carbon nanofiber (reprinted with permission from [138]; copyright 2015 Elsevier).

The poor electrical conductivity of the transition metal oxide anode materials is another inherent drawback that is preventing the commercialization of conversion anode materials. Many efforts have been made to form a composite with carbon that improves the electrical conductivity. Electrospinning and subsequent thermal treatment of a metal oxide and carbon precursor is the simplest technique to prepare the transition metal oxide/carbon composite anode material. Many phases of the transition metal oxides, such as Fe_3_O_4_ [107,108,133,134,135], Fe_2_O_3_ [111,126], Co_3_O_4_ [109,118,129], and CoO [136], were composited with carbon nanofibers. Figure 10a shows the Co_3_O_4_ porous carbon nanofiber synthesis process and the resulting product [109]. The Kirkendall and Ostwald ripening processes with incomplete thermal oxidation of carbon resulted in hollow Fe_2_O_3_ nanospheres surrounded with carbon (see Figure 10b) [116]. Electrodeposition on the carbon nanofiber is useful to fabricate core/shell structured carbon and transition metal oxide composites, and the morphologies of the deposited metal oxides are changed with the processing condition, such as the current density [137]. Figure 10c,d shows carbon nanofibers decorated with Fe_2_O_3_ nanorods and flower-like Co_3_O_4_ nanosheets [112,138]. The carbon composited metal oxide anodes exhibited improved electrochemical performances compared with the control groups.

**Figure 11:**
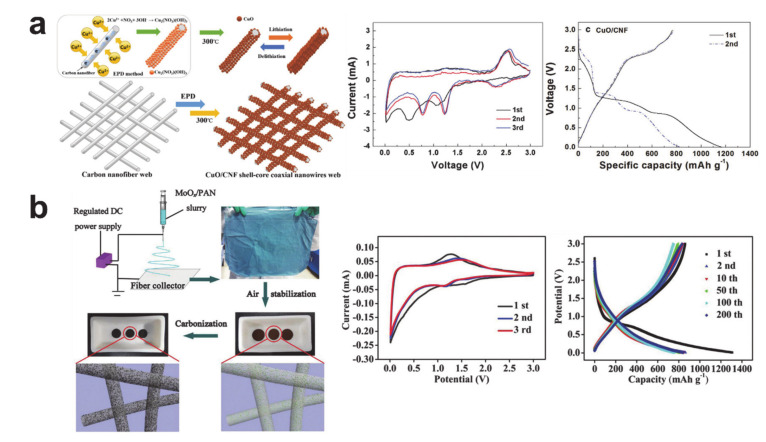
Fabrication process and electrochemical performances of other important carbon composite anode materials. (**a**) CuO/carbon composite nanofiber (reprinted with permission from [140]; copyright 2015 Springer Nature) and (**b**) MoO_2+δ_/carbon composite nanofiber (reprinted with permission from [145]; copyright 2016 Royal Society of Chemistry).

Other important transition metal oxide conversion materials are shown in Figure 11. Metallic copper is a current collector for the commercial lithium-ion battery anodes owing to its high electrical conductivity and electrochemical inactiveness when at the anode operating potential. On the contrary, copper oxide (CuO), which can store lithium via conversion reaction, has been researched as a high capacity anode material (theoretical capacity of 674 mAh/g) that is both environmentally friendly and cost-effective [139]. The electrochemical performance of raw CuO nanofiber [139] and CuO/carbon composite nanofiber [140] has been reported, and Figure 11a shows the electrodeposited CuO on carbon nanofiber and its electrochemical performance [140]. CVs of the CuO/carbon composite anode material exhibit the electrochemical reaction as follows: (i) lithium insertion at around 2 V to form Li_x_CuO, the partial reduction of copper to Cu^+^ and Li_2_O formation (CuO + 2Li^+^ + 2e^–^ → Cu_2_O + Li_2_O), SEI formation at around 1.1 V, and the further reduction of Cu^+^ to Cu^0^ and the formation of Li_2_O at around 0.5 V (Cu_2_O + 2Li^+^ + 2e^–^ → 2Cu + Li_2_O) during the first cathodic scan; (ii) the oxidation of copper from Cu^0^/Cu^+^ to Cu^2+^ to form CuO at around 2.5 and 2.7 V during anodic scans; and (iii) the reduction of copper from Cu^2+^ to Cu^+^/Cu^0^ at around 1.25 and 0.75 V during the following cathodic scans [139,140]. It is noteworthy that the fully reduced metallic Cu^0^ is highly irreversible, so the initial coulombic efficiencies of the CuO based nanofibers were lower than that of iron, cobalt, and nickel oxides.

Molybdenum oxide (MoO_2_) is an intriguing anode material because the insertion and conversion behaviors are co-exhibited in a certain condition. MoO_2_ has two different values of theoretical capacity: the theoretical capacity of bulk-sized MoO_2_ (insertion type) is 209 mAh/g, while nano-sized MoO_2_ (conversion type) is 838 mAh/g [141,142]. The insertion reaction is predominant with a fast electrochemical reaction and the conversion reaction is augmented with a slow electrochemical reaction because the cathodic reaction via conversion reaction occurs below 0.8 V [143]. Figure 11b shows the fabrication route and electrochemical performance of the conversion type MoO_2+δ_/carbon composite nanofiber. The CV curves demonstrate the following electrochemical reactions: (i) lithium insertion to form Li_0.98_MoO_2_, SEI formation, and the further reduction of Mo^+^ to Mo^0^ and the formation of Li_2_O during the first cathodic scan; and ii) a redox pair associated with the reversible phase transition of Li_x_MoO_2_ at the following scans [144,145]. Despite the highly reversible capacity of ca. 800 mAh/g being mainly attributed to the conversion reaction, the conversion reaction related plateau is not observed in the voltage profile.

**Figure 12:**
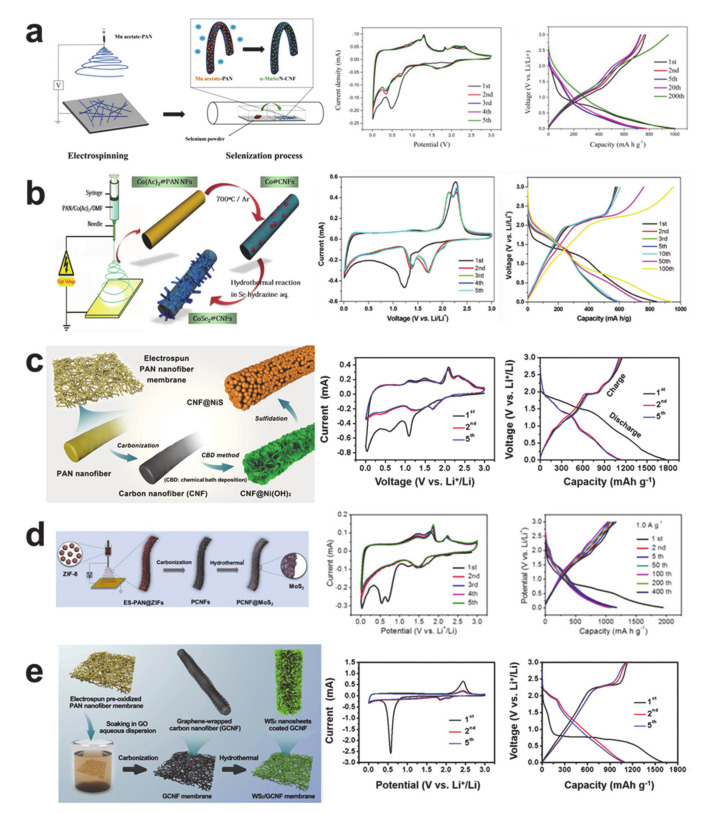
Transition metal chalcogenide anode materials. (**a**) α-MnSe/nitrogen-doped carbon composite nanofiber (reprinted with permission from [146]; copyright 2019 Elsevier), (**b**) CoSe_2_ nanorods on carbon nanofiber (reprinted with permission from [147]; copyright 2017 John Wiley and Sons), (**c**) NiS nanoparticles on carbon nanofiber (reprinted with permission from [148]; copyright 2015 John Wiley and Sons), (**d**) MoS_2_ nanoplates on porous carbon nanofiber (reprinted with permission from [150]; copyright 2019 Elsevier), and (**e**) WS_2_ nanosheets on carbon nanofiber (reprinted with permission from [151]; copyright 2016 Royal Society of Chemistry).

Transition metal chalcogenides (i.e., sulfides and selenides) can also be used as conversion anode materials via forming Li_2_S and Li_2_Se. Transition metal chalcogenide anode materials prepared by the electrospinning process are represented in Figure 12. The α-MnSe/nitrogen-doped carbon composite nanofiber in Figure 12a was synthesized through the electrospinning of the Mn/carbon precursor and in situ selenization during thermal treatment [146]. The CV curves show the SEI formation and the irreversible phase transition of α-MnSe crystal to β-MnSe by lithiation at round 0.5 V in the first cathodic scan, and a reversible redox reaction of Mn^0^/Mn^2+^ (oxidation at around 1.3 and 1.7 V and reduction at around 0.3 and 0.75 V) and Li_2_Se/Se (oxidation from 2 to 2.5 V and reduction at around 1.6 V). The voltage profiles show increased capacities from the initial reversible capacity of 769 mAh/g over time. The formation of CoSe_2_ nanorods on carbon nanofiber seen in Figure 12b was fabricated via electrospinning, thermal treatment, and a hydrothermal reaction [147]. The CV curves reveal the SEI formation, Li_2_Se formation, and reduction of Co^4+^ to Co^0^ in the first cathodic scan, and the redox reactions of Co^4+^/Co^0^ and Li_2_Se/Se (oxidations at 2.13 and 2.30 V and reductions at 1.72 and 1.37 V) in following cycles. Like the α-MnSe, the voltage profiles show an increase in specific capacity over time in comparison with the initial reversible capacity of 610 mAh/g. Figure 12c shows NiS nanoparticles attached to a carbon nanofiber [148]. In addition to the electrospinning and thermal treatment, a chemical bath deposition of Ni(OH)_2_ and sulfidation were carried out to synthesize the NiS nanoparticles onto the carbon nanofiber. The complex CV curves represent the stepwise nickel reduction to Ni^0^, Li_2_S formation, and the SEI formation in the first cathodic scan, and redox of the Ni^0^/Ni^2+^ and Li_2_S/S in the following scans. The voltage profiles show the high reversible capacity of 1149 mAh/g and the stability of the cycle behavior through repeated cycles.

Transition metal dichalcogenides such as MoS_2_ and WS_2_ are the most representative two-dimensional MXenes that have a graphene-like microstructure [149]. The MoS_2_ and WS_2_ may be misinterpreted as the intercalation anode materials because the microstructures are layered. MoS_2_ nanoplates and WS_2_ nanosheets on carbon nanofibers prepared via the hydrothermal process in Figure 12d,e are, however, able to demonstrate typical conversion electrochemical behaviors [150,151]. The CV curves of the MoS_2_ nanoplates on porous carbon nanofiber exhibit irreversible lithium intercalation to MoS_2_ (MoS_2_ + xLi^+^ + xe^–^ → Li_x_MoS_2_), SEI formation, and the complete reduction of molybdenum to Mo^0^ and the formation of Li_2_S in the first cathodic scan, as well as the reversible reduction of Mo^0^ to Mo^4+^, the partial reduction of Mo^4+^ to MO^6+^, and oxidation of Li_2_S to S in the anodic scan [152,153]. Likewise, the CV curves of the WS_2_ nanosheets on carbon nanofibers reveal the irreversible lithium intercalation to WS_2_ (WS_2_ + xLi^+^ + xe^–^ → Li_x_WS_2_), the SEI formation, and the complete reduction of tungsten to W^0^ and the formation of Li_2_S in the first cathodic scan, and reversible redox reactions of Li_2_S/S in the following scans [154,155]. Both the MoS_2_/carbon and WS_2_/carbon composite anode materials exhibited high reversible capacities (>1000 mAh/g) with stable voltage profiles after repeated cycles.

## 5. Alloying/Dealloying Reaction-Based Storage Materials

Anode materials that utilize the alloying reaction can potentially be used to create high energy density batteries thanks to their high theoretical capacity and relatively low working potential when compared with conversion anode materials. Examples include silicon (4212 mAh/g and <0.5 V) [156,157], tin (992 mAh/g and ≤0.8 V) [158,159], germanium (1626 mAh/g and <0.5 V) [160,161], and antimony (660 mAh/g and ca. 1.0 V) [162,163]. Structural deterioration, SEI formation, and electrical contact losses associated with the unavoidable large volume change during lithium insertion and extraction are the major drawbacks to using alloying anode materials.

**Figure 13:**
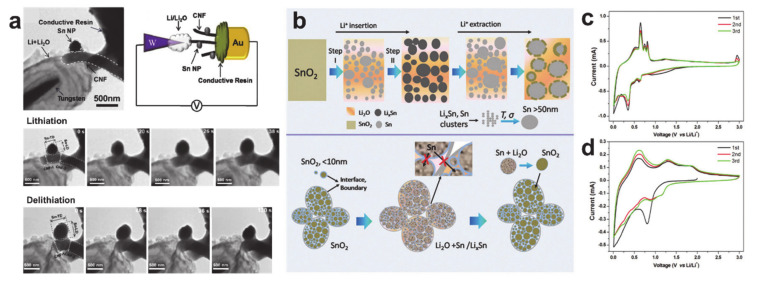
Electrochemical behaviors of Sn and SnO_2_. (**a**) Real-time observation of Sn alloying/dealloying behavior using in situ transmission electron microscopy (reprinted with permission from [176]; copyright 2015 Elsevier), (**b**) schematic illustrations of microstructural change of SnO_2_ during lithiation/delithiation (reprinted with permission from [177]; copyright 2016 Royal Society of Chemistry), and typical cyclic voltammetry curves of (**c**) Sn and (**d**) SnO_2_ crystals on porous carbon nanofibers (reprinted with permission from [178]; copyright 2016 Royal Society of Chemistry).

Despite the low theoretical storage capacity of tin, many tin precursors that can be used in the alloying process (SnCl_2_(∙2H_2_O) [164,165,166], SnCl_4_(∙5H_2_O) [167,168,169], Sn(Oct)_2_ [170,171,172], and Sn(OAc)_2_ [173]), as well as the carbothermal reduction of SnO_2_ into metallic Sn above 550 °C during thermal treatment [174,175,176], have encouraged intensive and extensive development of electrospun tin anode materials across a variety of tin compounds, from metallic Sn to amorphous SnO_x_. Real-time monitoring of metallic Sn on carbon nanofiber production in Figure 13a shows direct evidence of the alloying/dealloying mechanism (e.g., the volume change and crystalline structural change caused by formation of the polycrystalline Li_22_Sn_5_ alloy (lithiation) and polycrystalline Sn and Li_x_Sn (delithiation)) [176]. As explained in Section 2, the electrochemical reactions that occur in SnO_2_ are not as simple as those of the metallic Sn. Figure 13b shows the conversion and alloying/dealloying two-step lithiation/delithiation process and a hierarchical structural evolution of SnO_2_ during the electrochemical reactions [177]. The initial irreversible insertion process is prior to the conversion and alloying in the first discharge [35]. Although the reversibility of the conversion reaction (SnO_2_ + 4Li^+^ + 4e^−^ → Sn + Li_2_O) is thermodynamically unfavorable, the degree of reversibility can be increased to 95.5% by reducing the crystallite size of SnO_2_ [177]. Figure 13c,d shows the CVs of the metallic Sn and SnO_2_ formed on porous carbon nanofiber [178]. The CV curves of the metallic Sn on the porous carbon nanofiber exhibit the alloying/dealloying reactions: there is a prominent reduction attributed to Li–Sn alloying at around 0.35 V in the first cathodic scan, and the anodic scans show four distinct oxidation peaks at 0.52, 0.65, 0.75, and 0.81 V attributable to the dealloying of the Li_x_Sn alloys (e.g., Li_13_Sn_5_, Li_7_Sn_3_, LiSn, and Li_2_Sn_5_) [179,180,181]. The CV curves of SnO_2_ on the porous carbon nanofiber demonstrate the conversion and alloying/dealloying electrochemical reactions: reduction of tin to Sn^0^ by conversion reaction and SEI formation at around 0.8 V and Li–Sn alloying (Sn + xLi^+^ + xe^–^ ↔ Li_x_Sn (0 ≤ x ≤ 4.4)) in the first cathodic scan, and dealloying from Li_x_Sn at around 0.6 V and the reversible oxidation of Sn^0^ to Sn^2+^ (1.3 V) and Sn^2+^ to Sn^4+^ (1.9 V) during the anodic scans [172,182,183,184].

**Figure 14:**
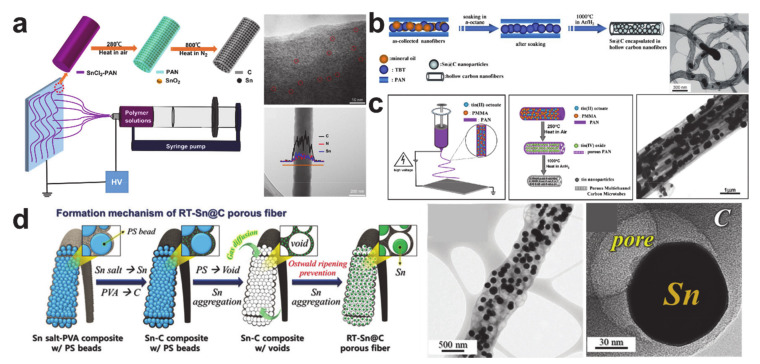
Processing routes and morphologies of metallic Sn/carbon composite anode materials. (**a**) Sn quantum dots/carbon composite nanofiber (reprinted with permission from [165]; copyright 2014 Elsevier), (**b**) Sn@C nanoparticles in hollow carbon nanofiber (reprinted with permission from [185]; copyright 2009 John Wiley and Sons), (**c**) Sn nanoparticles in multi-channeled carbon nanofiber (reprinted with permission from [187]; copyright 2009 American Chemical Society), and (**d**) rattle type Sn nanoparticles in porous carbon nanofiber (reprinted with permission from [188]; copyright 2018 Royal Society of Chemistry).

The key issues that require attention when using metallic Sn are the pulverization and aggregation processes that occur during electrochemical reactions [185]. As such, confining the Sn nanoparticles to a carbonaceous matrix is the best option to prevent the aggregation of the Sn particles and the electrical contact loss that occurs owing to pulverization. A small particle size and uniformity in the Sn nanoparticles are important to avoid fracture, as is the ability of the matrix to provide a buffer space for the volume expansion [186]. Figure 14 shows the Sn nanoparticle/carbon nanofiber composites with the above requirements satisfied. The monodispersed Sn particles (<10 nm) embedded in a nitrogen-doped carbon nanofiber in Figure 14a exhibited excellent electrochemical performance as a result of the synergistic effect of the Sn quantum dots and nitrogen-doped porous carbon matrix [165]. Design of void space becomes more important if the diameter of the Sn nanoparticles is greater than 10 nm. Sn/C composite nanofibers engineered to have a larger void space are shown in Figure 14b–d. The bamboo-like composite of Sn@C nanoparticles in a hollow carbon nanofiber shown in Figure 14b was fabricated using the coaxial electrospinning process with mineral oil as a sacrificial material [185], while the Sn nanoparticles dispersed in a multi-channeled carbon nanofiber shown in Figure 14c were synthesized using the single-nozzle electrospinning process and a pore generation polymer (poly(methyl methacrylate) (PMMA)) [187]. Rattle-like Sn nanoparticles in a porous carbon nanofiber, shown in Figure 14d, were created using void generation polymers (polystyrene (PS) nanobeads) and a controlled thermal treatment [188]. The Sn nanoparticles in the void engineered carbon nanofibers commonly exhibited excellent cycling performances over a hundred cycles as a result of advantageous structures.

**Figure 15:**
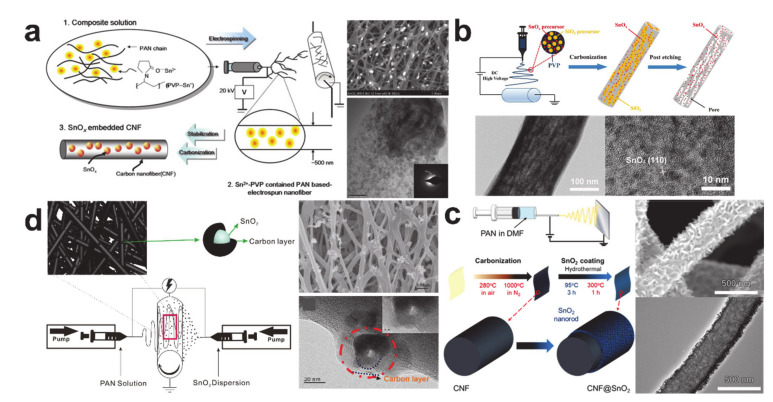
Processing routes and morphologies of SnO_x_/carbon composite anode materials. SnO_x_ nanoparticle containing carbon nanofibers with (**a**) microporous microstructure (reprinted with permission from [173]; copyright 2011 Elsevier) and (**b**) mesoporous microstructure (reprinted with permission from [190]; copyright 2016 American Chemical Society), and carbon nanofibers decorated with (**c**) carbon-coated SnO_2_ nanoparticles (reprinted with permission from [191]; copyright 2019 Elsevier) and (**d**) SnO_2_ nanorods (reprinted with permission from [192]; copyright 2018 American Chemical Society).

Tin oxides have been an active area of research owing to their higher theoretical capacities than metallic tin (SnO (1273 mAh/g) and SnO_2_ (1494 mAh/g)), which are attributable to their conversion and alloying/dealloying electrochemical reactions [184]; however, SnO_x_ compounds still suffer from a large volume expansion and poor cycling stability [189]. Two types of tin oxide and carbon composites have been proposed that remove the disadvantages associated with tin oxides. The first strategy is simply to embed SnO_x_ nanoparticles into micro and mesoporous carbon nanofibers, as shown in Figure 15a,b, similar to the design rationale used in the creation of metallic tin and carbon composites [173,190]. Anchoring SnO_2_ nanostructures, such as carbon coated SnO_2_ nanoparticles and SnO_2_ nanorods, to the carbon nanofiber surface in Figure 15c,d is the second strategy, which focuses on improving lithium-ion transport [191,192]. The composites from the first approach showed better cycling performance than those that used in the second approach, leading to the conclusion that tin oxide embedded into a void engineered carbon matrix is a better energy storage structure than tin oxide anchored onto the surface of a carbon structure.

**Figure 16:**
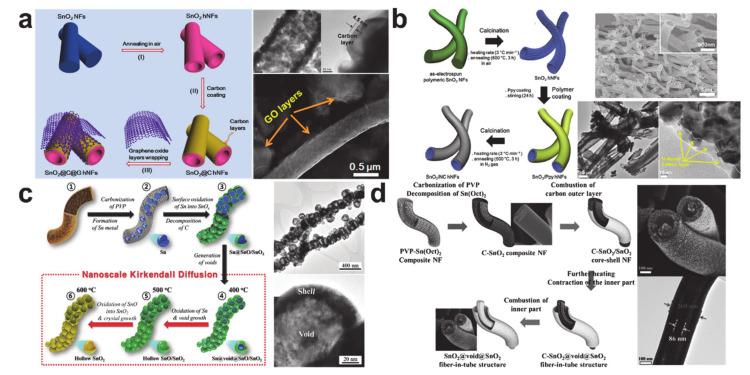
Hollow structured SnO_2_ anode materials. (**a**) Carbon-coated and graphene oxide (GO) layer wrapped SnO_2_ hollow nanofiber (reprinted with permission from [193]; copyright 2015 Elsevier), (**b**) nitrogen-doped carbon-coated SnO_2_ hollow nanofiber (reprinted with permission from [194]; copyright 2017 Elsevier), (**c**) nanofiber composed with hollow SnO/SnO_2_ nanospheres (reprinted with permission from [195]; copyright 2015 John Wiley and Sons), and (**d**) fiber-in-tube structured SnO_2_ nanofiber (reprinted with permission from [196]; copyright 2014 John Wiley and Sons).

Hollow-structured SnO_2_ nanostructures have been designed for both a short lithium-ion diffusion length and a void space that is able to accommodate the volume change that occurs during electrochemical reactions. Ostwald ripening-driven hollow structure formation during calcination was employed to synthesize hollow SnO_2_ nanostructures. The carbon-coated hollow SnO_2_ nanofibers shown in Figure 16a,b exhibited an improved electrochemical performance compared with the hollow SnO_2_ nanofiber without a carbon coating layer, with the improvement being generated by suppressing SEI formation and providing electrical pathways [193,194]. The optimized and well-defined hollow SnO_2_ nanostructures based on the Kirkendall diffusion shown in Figure 16c,d showed excellent cycling performance over 250 cycles as well as excellent rate performance at high rates (≥5000 mA/g) [195,196]. The combination of structural optimization and composites with a conducting shell is regarded as the best option when designing the most effective tin-based anode material.

**Figure 17:**
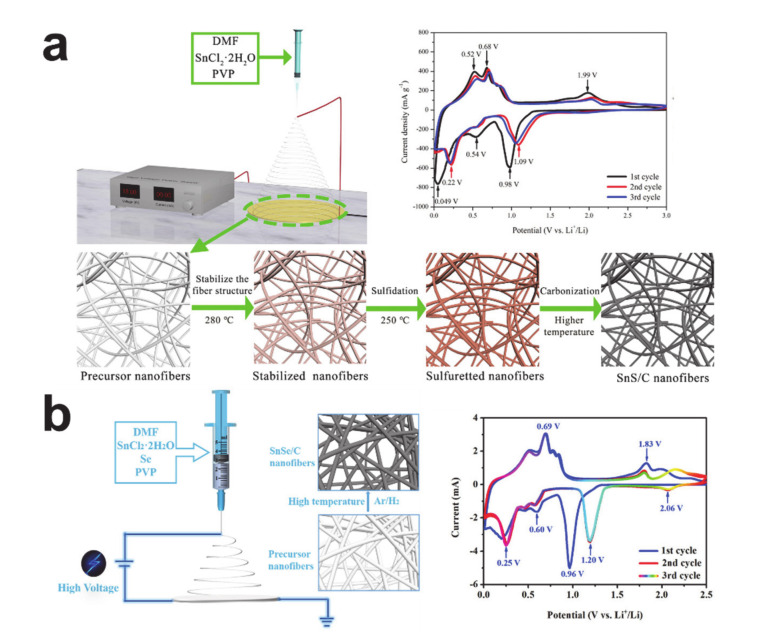
Tin chalcogenide/carbon composite anode materials. (**a**) SnS/carbon composite nanofibers (reprinted with permission from [199]; copyright 2019 Elsevier) and (**b**) SnSe/carbon composite nanofiber (eprinted with permission from [200]; copyright 2020 Elsevier).

Tin chalcogenides are another tin-based anode material that stores lithium via both the conversion and alloying electrochemical reactions. The high theoretical capacity of tin sulfide (SnS, 1137 mAh/g) is attributed to both the conversion (SnS + 2Li^+^ + 2e^−^ ↔ Sn + Li_2_S) and alloying/dealloying reaction (Sn + 4.4Li^+^ + 4.4e^−^ ↔ Li_4.4_Sn) [197,198]. The schematic synthesizing routes and CV curves of SnS and SnSe/carbon composite nanofibers are shown in Figure 17. The SnS/carbon composite nanofiber in Figure 17a was synthesized via electrospinning and subsequent heat treatment [199]. Sulfidation was conducted in between the oxidative stabilization and carbonization stages to provide sulfur and to form SnS crystals. The reversible capacity of the SnS/carbon composite nanofiber was 898 mAh/g. The CV curves of SnS/carbon composite nanofiber exhibit the electrochemical reactions of SnS as follows: (i) the conversion reaction of SnS (SnS + 2Li^+^ + 2e^−^ ↔ Sn + Li_2_S) at 0.98 V, the SEI formation at 0.54 V, and the alloying reaction (Sn + xLi^+^ + xe^–^ ↔ Li_x_Sn (0 ≤ x ≤ 4.4)) below 0.3 V observed in the first cathodic scan; (ii) two peaks of multi-step de-alloying of Li-Sn at 0.52 V and 0.68 V seen in the anodic scan; and (iii) the oxidation/reduction peaks located at 1.99 V/1.09 V attributed to the reversible conversion reaction. The theoretical capacity of tin selenide (SnSe, 847 mAh/g) is not greater than that of metallic tin, despite the similar electrochemical reactions (SnSe + 2Li^+^ + 2e^−^ ↔ Sn + Li_2_Se (conversion) and Sn + 4.4Li^+^ + 4.4e^−^ ↔ Li_4.4_Sn (alloying/dealloying)), because of the high atomic weight of the selenium [198]. The SnSe/carbon composite nanofiber in Figure 17b was prepared by electrospinning selenium containing a precursor solution and a subsequent thermal treatment under a reducing atmosphere (Ar/H_2_ mixture) [200]. The reversible capacity of the SnSe/carbon composite nanofiber was 685 mAh/g. The CV curves represent the electrochemical reactions of the SnSe: (i) the conversion reaction of SnSe (SnSe + 2Li^+^ + 2e^−^ ↔ Sn + Li_2_Se) at 0.96 V (at 1.20 V in the following scans), the SEI formation, and the alloying reaction (Sn + xLi^+^ + xe^–^ ↔ Li_x_Sn (0 ≤ x ≤ 4.4)) at 0.6 V and below in the first cathodic scan; (ii) a few peaks showing the multi-step de-alloying of Li-Sn at around 0.68 V in the anodic scan; and (iii) the oxidation peaks located at 1.83 V and above originating from the reversible conversion reaction. It is noteworthy that the tin chalcogenides are currently being researched as potential material candidates for use with sodium and potassium ion battery anodes [201,202,203].

Without doubt, silicon is the most important alloying anode material owing to its extraordinarily high specific capacity (4212 mAh/g) and low working potential (<0.5 V) [156,157]. The large volume expansion (~400%) during lithiation, pulverization, and subsequent electrical contact loss are very well-known disadvantages of silicon-based anode materials. Note that the practical capacity (3579 mAh/g) and volume change (280%) are lower than the theoretical values owing to the formation of Li_3.75_Si phase by lithiation [204]. Despite the disadvantages, the merits of silicon are much more significant than any other conversion and alloying anode materials. Therefore, a large number of battery researchers and the leading global battery manufacturers, such as Samsung SDI and LG Chem, have put tremendous efforts into the development and commercialization of silicon anode materials. Although some technologies have been integrated into commercial lithium rechargeable batteries, the composition of silicon in a commercial lithium-ion battery anode is only about 5%. Thus, there is still the potential for silicon anode material development that can increase the energy density of the commercial lithium-ion battery.

**Figure 18:**
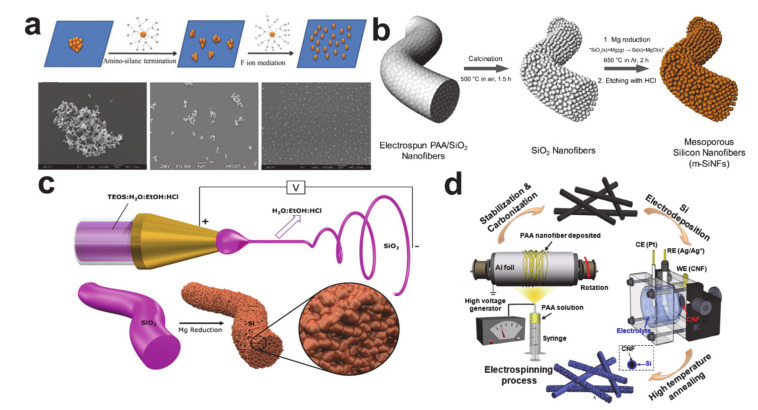
Schematic illustrations of various silicon sources and processing routes. (**a**) De-agglomerated Si nanoparticles (reprinted with permission from [211]; copyright 2014 Elsevier), (**b**) SiO_2_ nanoparticle-based mesoporous silicon nanofiber (reprinted with permission from [212]; copyright 2013 American Chemical Society), (**c**) tetraethyl orthosilicate-based silicon nanofiber (reprinted with permission from [216]; copyright 2015 Springer Nature), and (**d**) SiCl_4_-based Si-coated carbon nanofiber (reprinted with permission from [218]; copyright 2014 Elsevier).

There are a wide variety of silicon sources that each require different processing routes (see Figure 18). When synthesizing Si-based composite materials, it is preferable to use Si nanoparticles directly for heat treatment, as the nanoparticles are chemically resistant up to temperatures of 1000 °C under inert gases [205] and, therefore, Si nanoparticle-based composite nanofiber anode materials have been frequently studied [206,207,208,209,210]. The de-agglomeration of the Si nanoparticles via amino-silane functionalization and fluoride-ion mediation shown in Figure 18a can be used to avoid unsatisfactory cyclic performance of the electrodes [211]. SiO_2_ nanoparticles have also been used as silicon sources in the formation of mesoporous microstructures via magnesiothermic reduction and the subsequent etching of MgO, as shown in Figure 18b [212]. Tetraethyl orthosilicate (TEOS) has also been employed as a liquid precursor in the manufacture of well-defined silicon nanostructures [213,214,215]. Initially formed SiO_2_ precursor nanostructures are transformed to the Si nanostructures via magnesiothermic reduction and selective MgO etching (see Figure 18c) [216]. Dry and wet chemical deposition processes were enabled by tetrahedral silicon compounds: SiH_4_ was used as a chemical vapor deposition source to coat silicon on the carbon nanofiber [217], while SiCl_4_ was employed in the electrodeposition of silicon on the carbon nanofiber, as shown in Figure 18d [218]. Note that the typical electrochemical reactions are assigned as follows: SEI formation at around 0.7 V and Li–Si alloy formation below 0.1 V during the first lithiation stage, dealloying from Li–Si alloy at 0.33 and 0.47 V during delithiation, and Li–Si alloy formation at around 0.2 V during the following lithiation processes [219,220,221].

The ability to use pure silicon as a single anode material is highly desired in the design of high energy density batteries. Unfortunately, challenges associated with the large volume expansion are not easy to resolve. This has led to many speculative and novel silicon-based materials being proposed to enable silicon to be used in commercial batteries. Most of these efforts have been focused on silicon oxides (SiO_x_) and silicon/carbon composites. The silicon oxides (SiO_x_) are expected to maintain long-term stable cycling performances as they can effectively accommodate the volume change of Si to SiO_2_ matrix into the SiO_x_ structure [222]. The major disadvantages associated with the materials are a low electrical conductivity, low lithium mobility, and a huge irreversible capacity loss owing to the formation Li_2_O and Li_4_SiO_4_ from the SiO_2_ phase [223]. Carbonaceous matrices, however, especially electrospun carbon nanostructures, show excellent material properties (e.g., electrical conductivity: 1.95 to 7.69 S/cm, modulus: 80 to 191 GPa, and strength: 1.86 to 3.52 GPa) [224]. In addition, the fabrication of the carbon nanofiber is a well-established process of electrospinning and thermal treatment. Thus, a number of silicon/carbon composite nanofibers have been reported because of processing convenience and excellent material properties of carbon nanofibers.

**Figure 19:**
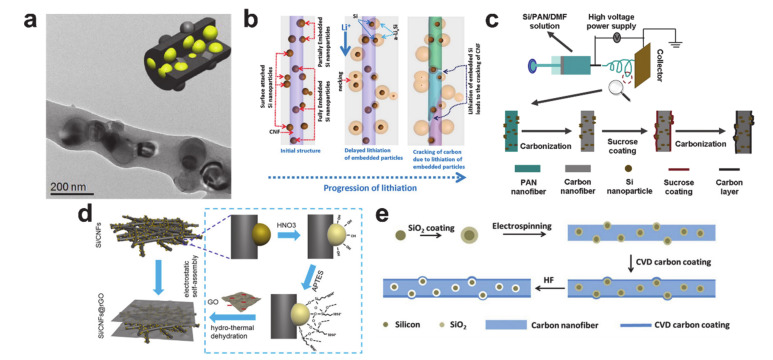
Silicon nanoparticles embedded carbon nanofiber anode materials. (**a**) Schematic diagram and morphology of carbon nanofiber containing silicon nanoparticles (reprinted with permission from [211]; copyright 2014 Elsevier), (**b**) schematic illustrations of morphological changes by lithiation (reprinted with permission from [225]; copyright 2012 American Chemical Society), (**c**) carbon-coated silicon/carbon composite nanofiber (reprinted with permission from [226]; copyright 2015 Elsevier), (**d**) graphene-protected silicon/carbon composite nanofiber (reprinted with permission from [227]; copyright 2016 Elsevier), and (**e**) chamber-confined silicon/carbon composite nanofiber (reprinted with permission from [228]; copyright 2014 Royal Society of Chemistry).

A schematic illustration and morphology of typical silicon nanoparticles contained in carbon nanofibers are shown in Figure 19a [211]. The main role of the silicon nanoparticles is to store a large amount of lithium, whereas the carbon matrix performs multiple roles: (i) to protect the silicon from the electrolyte, (ii) to provide electronic and ionic pathways, and (iii) to withstand the mechanical stress induced from the volume expansion. The carbon matrix is not always perfect at fulfilling these roles. As shown in Figure 19b, the carbon matrix can be broken by the mechanical stress from the silicon volume expansion [225]. It is important to prevent the sudden breaking of the carbon matrix to maintain electrochemical performance for as long as possible. To prevent failure of the silicon/carbon composite nanofibers, various approaches have been considered: (i) an additional carbon coating on the silicon/carbon composite nanofiber to withstand the silicon volume expansion and to prevent direct contact of the silicon with the electrolyte (Figure 19c) [226], (ii) introducing the reduced graphene oxide on the silicon/carbon composite nanofiber mat for the same purpose as the carbon coating (Figure 19d) [227], and (iii) confined vacant space formation surrounding the silicon nanoparticles in the silicon/carbon composite nanofiber (Figure 19e) [228]. The carbon protection coating and vacant space formation improved both the cycle and rate performances by delaying carbon matrix failure.

**Figure 20:**
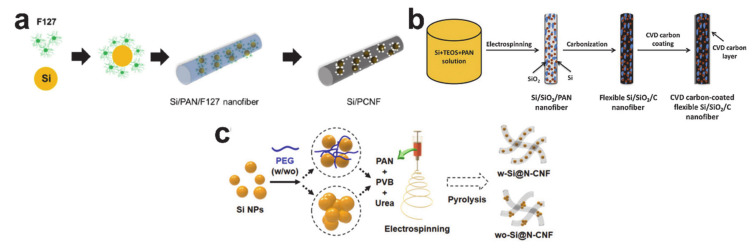
Functional additive introduced silicon/carbon composite nanofibers. (**a**) Pluronic F127 containing precursor driven silicon/porous carbon composite nanofiber (reprinted with permission from [230]; copyright 2015 Elsevier), (**b**) carbon-coated silicon/silica/carbon composite nanofiber (reprinted with permission from [231]; copyright 2015 Elsevier), and (**c**) polyethylene glycol (PEG) containing precursor driven carbon nanofiber containing uniformly dispersed silicon nanoparticles (reprinted with permission from [232]; copyright 2017 Elsevier).

Further work has been undertaken with functional additives to improve the silicon/carbon composite nanofiber structures. Carbon nanotubes (CNTs) were added into the precursor solution to fabricate the silicon/CNT/carbon composite nanofiber [229]. The CNTs reduced the volume expansion of silicon nanoparticles and improved the mechanical stability of the electrode. Figure 20a shows the silicon/porous carbon composite nanofiber formed from the Pluronic F127 containing precursor solution [230]. An amphiphilic surfactant (Pluronic F127) was introduced to disperse the silicon nanoparticles and as a template to form a porous structure to absorb the volume expansion. The silicon/silica/carbon ternary composite nanofiber in Figure 20b was made from a precursor solution mixed with TEOS [231]. This TEOS driven nanoscale silica gave the composite nanofiber extra flexibility that reduced the mechanical stress deriving from silicon volume expansion. Low molecular weight polyethylene glycol (PEG) was used as the sole de-agglomerating agent to fabricate a well-dispersed silicon/carbon composite nanofiber (Figure 20c) [232]. The electrochemical performance of the silicon/carbon composite nanofibers with functional additives was improved when compared with composite nanofibers without additives. However, the cycle performance improvements are not significant enough to make commercial production of a simple mixed configuration worthwhile.

**Figure 21:**
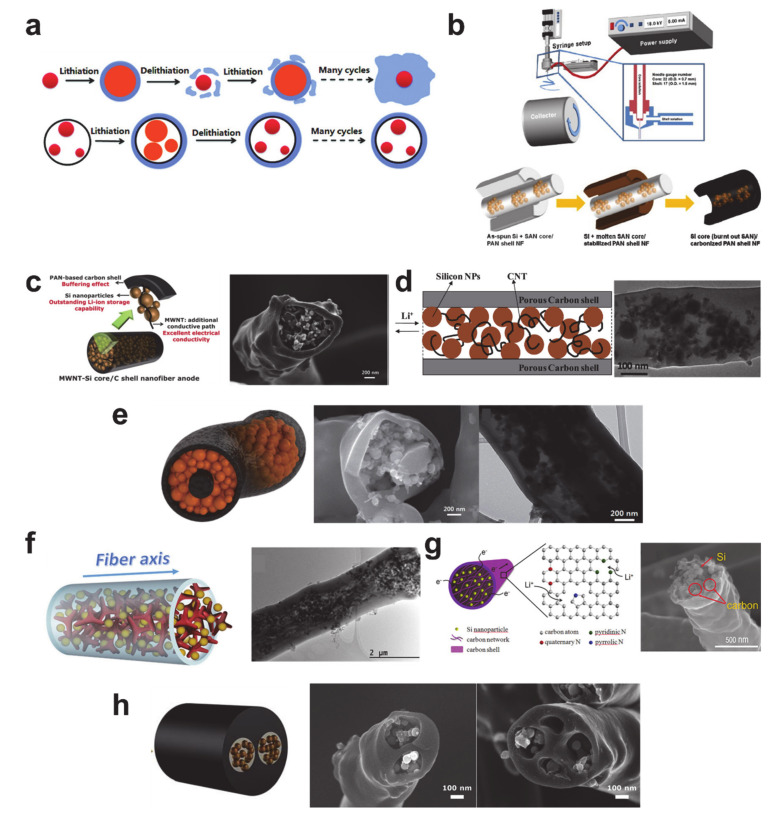
Hollow carbon nanofiber anode materials containing silicon nanoparticles in void space. (**a**) Schematic diagram of structural change and solid electrolyte interphase growth on the raw silicon and the silicon contained in hollow carbon structure (reprinted with permission from [233]; copyright 2012 American Chemical Society) and coaxially electrospun silicon/carbon composite nanofibers: (**b**) silicon core/carbon shell composite nanofiber (reprinted with permission from [234]; copyright 2012 Elsevier), (**c**) silicon-carbon nanotube core/carbon shell composite nanofiber (reprinted with permission from [235]; copyright 2013 Royal Society of Chemistry), (**d**) silicon-carbon nanotube core/carbon shell composite nanofiber (reprinted with permission from [236]; copyright 2014 Royal Society of Chemistry), (**e**) carbon core/silicon medium/carbon shell composite nanofiber (reprinted with permission from [238]; copyright 2014 Royal Society of Chemistry), (**f**) silicon-carbon core/carbon shell composite nanofiber (reprinted with permission from [239]; copyright 2015 Royal Society of Chemistry), (**g**) silicon-carbon core/carbon shell composite nanofiber (reprinted with permission from [240]; copyright 2019 Elsevier), and (**h**) multi-channeled silicon core/carbon shell composite nanofiber (reprinted with permission from [205]; copyright 2014 Royal Society of Chemistry).

It is clear that mechanical failure of the carbon matrix is inevitable without void engineering. As a result, void space has been introduced to provide room for silicon volume expansion to prevent the mechanical failure of the carbon matrix and also to improve the electrochemical performance of the silicon/carbon composite nanofibers. Hollow carbon nanofibers containing silicon nanoparticles are the best structures for superior cycling performance as they are able to accommodate the silicon volume change and also protect the silicon from SEI formation (Figure 21a) [233]. Hollow carbon nanofibers containing silicon nanoparticles in the void space are easily manufactured by the coaxial electrospinning process. The silicon core/carbon shell composite nanofiber in Figure 21b was fabricated using SAN as a void generation polymer [234]. The composite nanofiber exhibited a stable cycling performance up to 50 cycles with a reversible capacity of 596 mAh/g. There are multiple studies on silicon core/carbon shell composite nanofiber anode materials, in which the amount of silicon is increased and the electrical contact is increased through the addition of carbon shells and auxiliary carbon additives, resulting in materials that have a specific capacity greater than 1000 mAh/g and stable cycling performances. Examples include the following: (i) silicon–CNT core/carbon shell composite nanofibers, shown in Figure 21c,d [235,236,237]; (ii) carbon core/silicon medium/carbon shell composite nanofibers in Figure 21e [238]; (iii) a silicon core with pyrolyzed carbon/carbon shell composite nanofibers in Figure 21f,g [239,240]; and (iv) multi-channeled silicon core/carbon shell composite nanofibers in Figure 21h [205]. In addition to structural optimization, the addition of an auxiliary anode material can help to improve the electrochemical performance of the silicon/carbon composite nanofibers. Separately compartmentalized silicon and tin nanoparticles in double-holed carbon nanofibers were shown to achieve an exceptional rate performance by scavenging the lost Si charge capacity at higher working potentials of Sn under increased current densities without sacrificing any other electrochemical performances [241]. Though the electrochemical performances of these materials have not been fully studied, and are thus not ready to be implemented in commercial batteries, it is expected in future that these materials will be commercialized after minor structure and property optimization.

Other alloying anode materials that possess high theoretical capacities such as germanium (1626 mAh/g for Li_4.4_Ge) [160,161] and phosphorous (2595 mAh/g for Li_3_P) [242] are also viable candidates for high energy density lithium-ion batteries. It may be a simpler option to develop the germanium- and phosphorous-based anode materials, as some processing technologies for advanced silicon anode materials can be directly applied to these materials. Nevertheless, performance and feasibility should be examined prior to development owing to the relatively inferior lithium storage capacities of the elements.

## 6. Plating/Stripping-Based Storage Materials

Lithium metal with a high specific capacity (3860 mAh/g) and the lowest working potential of the materials discussed (−3.04 V vs. the standard hydrogen electrode (SHE)) has recently been revisited as a highly desirable anode material when pursuing huge energy densities (e.g., 3505 Wh/kg for Li–O_2_ batteries and 2600 Wh/kg for Li–S batteries) [243,244]. The large volume change observed during the electrochemical reaction and dendrite growth are the primary issues of the lithium metal anode. The dendrite growth causes secondary issues such as continuous SEI formation, dead lithium formation, and electrolyte depletion. Moreover, the dendrite growth has also been well-known as an origin of battery fires, caused by short-circuiting and subsequent thermal runaway. Therefore, suppressing the volume change and dendrite formation is the most important task in lithium metal battery development. Electrospun nanofiber mats are attractive lithium plating/stripping anodes, as their porous structures minimize the volume change and the large specific area of the electrospun mats reduces the current densities. Thus, current research has been focused on improving high performance lithium metal anodes via introducing lithophilic surfaces and regulating lithium deposition behavior.

**Figure 22:**
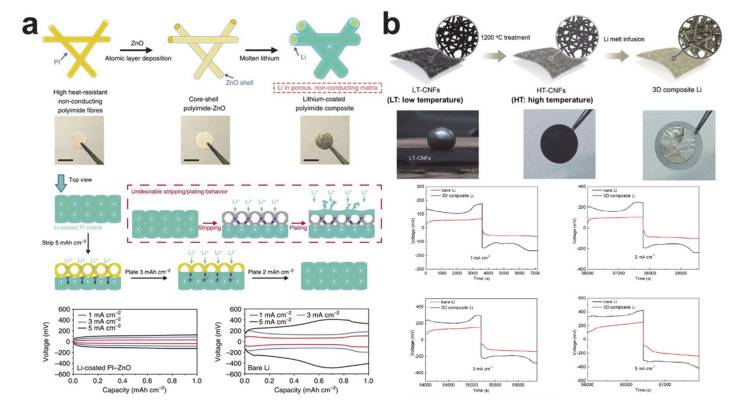
Lithium/nanofiber mat composite electrodes and their electrochemical performances. (**a**) Lithium/zinc oxide coated polyimide composite (reprinted with permission from [245]; copyright 2016 Springer Nature) and (**b**) lithium/carbon composite (reprinted with permission from [247]; copyright 2017 Royal Society of Chemistry).

The thickness of an anode can be changed by tens of microns when using a lithium metal anode; a deposition of bare lithium of 1 mAh/cm^2^ corresponds to a lithium thickness of 4.85 μm [245]. The scaffold or host structure is necessary to reduce the volume change of the lithium metal anode cell on lithiation. The thickness of the nanofiber mat is controlled by the processing time and, in simple terms, a thicker mat can increase the lithium uptake capacity. A lithium/scaffold composite can be easily formed by infusing molten lithium metal into the scaffold, as the melting temperature of lithium is 180 °C [246]. The issue is contact between lithium and the nanofiber mat, and lithophilic nanofiber surface formation has been used to attempt to make the contact intimate (Figure 22). Zinc oxide was coated on the polyimide (PI) surface using atomic layer deposition to render the matrix wet with molten lithium, as shown in Figure 22a. The composite electrode exhibited little volume change during lithium plating and stripping and had a lower overpotential compared with lithium at all current densities. Electrochemically active carbon nanofibers were also used as the host for the composite electrodes. Interestingly, the lithiophilicity of carbon nanofibers varied with the carbonization temperature because of the difference in the surface carbonaceous microstructures (see Figure 22b) [247]. The carbon/lithium composite also showed an improved electrochemical performance compared with that of bare lithium. The silicon coating layer was used to make the carbon nanofiber mat lithophilic [248]. Likewise, the composite electrode exhibited a low volume change as well as a reduced overpotential during electrochemical reactions.

**Figure 23:**
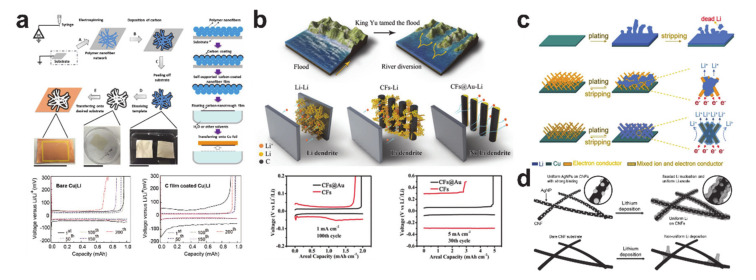
Nanofibrous lithium metal hosts and their electrochemical performances. (**a**) Semi-tubular carbon film (reprinted with permission from [250]; copyright 2017 Elsevier), (**b**) gold-coated carbon nanofiber (reprinted with permission from [251]; copyright 2018 John Wiley and Sons), (**c**) Li_6.4_La_3_Zr_2_Al_0.2_O_12_ nanoparticle incorporated carbon nanofiber (reprinted with permission from [252]; copyright 2018 John Wiley and Sons), and (**d**) silver nanoparticles decorated carbon nanofiber (reprinted with permission from [253]; copyright 2017 John Wiley and Sons).

Current lithium-ion batteries are operated only with the lithium initially contained in the cathode material. In other words, there is no additional source of lithium other than the cathode. This means that the lithium metal host does not have to contain lithium if the ideal case is assumed and the reaction is reversible. However, electrochemical performances of real lithium-free anode cells deteriorate owing to the SEI formation and dead lithium formation with non-uniform lithium plating and stripping [249]. Thus, regulating the lithium deposition behavior is crucial to improve the rechargeability of the lithium metal anodes. Figure 23 shows various designs of the lithium plating scaffold produced from electrospun nanofibers. The semi-tubular carbon film in Figure 23a was synthesized by templating an electrospun PVP nanofiber mat [250]. The carbon film guided homogenous lithium to be deposited underneath the film in the absence of Li dendrites. As a result, overpotentials of the carbon film-coated Cu|Li asymmetrical cell after the first cycle were much lower than those of the bare Cu|Li cell. Research has been undertaken on more delicate mechanisms to control the lithium plating behavior. As shown in Figure 23b, a gold coating layer was employed on the back side (the side away from the separator) to encourage selective deposition of lithium ions through homogeneous seeded growth using gold nanoparticles as seeds [251]. The gold-coated carbon nanofibers exhibited a lower overpotential and longer cyclability. Likewise, a mixed ion- and electron-conducting network consisting of a superionic conducting material (Li_6.4_La_3_Zr_2_Al_0.2_O_12_ (LLZO)) and an excellent electronic conducting material (carbon) was designed for homogeneous plating and rapid stripping of lithium (Figure 23c) [252], and ultrafine silver nanoparticles with a carbon composite nanofiber were synthesized using the Joule heating method to guide preferential lithium plating on the carbon nanofiber (Figure 23d) [253]. Despite such impressive progress on the plating/stripping anode materials, there are still significant margins of improvement in advancing lithium metal batteries.

## 7. Current Limitations and Prospects

Firstly, the legacy of electrospun nanofiber anode materials must be used to contribute to the improvement of commercial lithium-ion batteries. There should be an appreciation for the progress made in electrospun nanofiber anode material development; novel concepts, optimum structures, and significant performance improvements have been made via developments in the electrospinning process. On the basis of current progress, it is now viable to implement nanofiber-based anode materials into conventional lithium-ion batteries, primarily owing to developments in insertion, conversion, and alloying anode materials that are proven and ready. There are many papers showing results that target and offer improvements to current commercial lithium-ion batteries, such as higher energy densities, higher power densities, and satisfactory rapid charging performances. However, a large number of parameters must meet the specification requirements for a new electrode material to be implemented in commercial batteries; for example, the loading level (areal specific active material weight), electrode porosity and density, electrode volume change, initial coulombic efficiency, long-term cyclability, high rate performance, operating temperature, and so on. It is extremely difficult to satisfy all the specification requirements; therefore, it may be more reasonable to make small improvements by adding electrospun nanofiber anode materials to existing graphite electrode production.

Secondly, greater effort needs to be put into developing lithium metal anodes. The lithium metal anode is the ultimate anode system enabling super-high energy density. As previously mentioned, using a porous electrospun mat is structurally beneficial to storing lithium by plating, and the wide variety of material species that can be applied to the product is advantageous. There are many great opportunities to develop lithium metal anodes using the electrospinning process.

Thirdly, the acceleration of solid-state battery development can be encouraged through active studies of nanofiber anode materials. Active research is being conducted on solid-state batteries to consider factors such as enhancing the safety and improving the energy density of conventional lithium-ion batteries by utilizing organic electrolytes [254]. However, very few studies are being conducted on the nanofiber-based solid-state battery anodes [186,255,256]. Identifying the optimum electrode materials and structures should be highly encouraged as this may unearth a totally different ionic transport mechanism, and different interfacial interactions between electrolytes and active materials or cell fabrication processes. The electrospinning process and resultant nanofiber can provide a screening platform to optimize the active material and electrode structure for solid-state battery anodes.

## 8. Concluding Remarks

Research has progressed on nanofiber-based anode materials that utilize each of the electrochemical reaction types, from insertion (or intercalation) to plating. Electrospun anode materials were designed to improve the desired properties and to reduce the negative properties based on the electrochemical reaction type being studied. Some of the more prominent achievements are already at a high technology-readiness level and are ready to be implemented in commercial lithium-ion battery production; furthermore, forming a graphite composite with the existing electrode can reduce the difficulty barrier to introducing nanofiber-based anode materials. More effort is required with regard to promoting the use of anode materials for lithium metal batteries and solid-state batteries, in order to accelerate next-generation battery development.
